# How Conscientiousness Influences Prosocial Behavior Among Adolescents: The Role of Empathy and Social Support

**DOI:** 10.3390/bs16030468

**Published:** 2026-03-21

**Authors:** Weina Lei, Xiaogang Xia, David Yun Dai, Guihua Wang

**Affiliations:** 1 Department of Educational and Psychological Science, Yuncheng University, Yuncheng 044000, China; 2 School of Education, Shaanxi Normal University, Xi’an 710062, China; xiaxiaogang@snnu.edu.cn; 3 Educational Psychology and Methodology, University at Albany—SUNY, Albany, NY 12222, USA; ydai@albany.edu; 4 Department of Chinese, Yuncheng University, Yuncheng 044000, China; wangguihua@ycu.edu.cn

**Keywords:** conscientiousness, empathy, social support, prosocial behavior, adolescents

## Abstract

Existing research highlights the positive relationship between conscientiousness and prosocial behavior. Yet the underlying psychological mechanism between these variables needs further exploration. This study investigated the mediating role of empathy and the moderating role of social support in the relationship between conscientiousness and prosocial behavior by constructing a moderated mediation model. The study included 1081 middle school students from China, aged 13 to 18 years (*M* = 15.45, *SD* = 1.91). The sample consisted of 531 boys (49.12%) and 550 girls (50.88%), all of whom completed surveys on conscientiousness, empathy, social support, and prosocial behavior. Results revealed that empathy partially mediated the relationship between conscientiousness and prosocial behavior, while social support moderated both the direct relationship between conscientiousness and prosocial behavior and the indirect relationship between conscientiousness and prosocial behavior mediated by empathy. The findings provide educational implications for cultivating prosocial behavior among adolescents.

## 1. Introduction

Prosocial behavior is broadly defined as positive and voluntary behaviors or actions aimed at benefiting other individuals, groups, or communities, which can be identified as kindness, assistance, generous donations, caring for others, helping disadvantaged groups, participating in community services and demonstrating concern about social developments (Eisenberg & Miller, 1987; Eisenberg, 2006; Scourfield et al., 2004). Studies have demonstrated that prosocial behavior has positive impacts on adolescents’ psychological development. For instance, adolescents with more prosocial behaviors are likely to show better adaptability and greater wellbeing, lower aggression and less bullying behavior (X. Fu et al., 2023; Gregori et al., 2025; Hu et al., 2021; Wentzel, 2014). In addition, increases in adolescents’ prosocial behavior are also beneficial for fostering more emotional support and academic engagement, which contribute to academic success (Gerbino et al., 2018; Guo et al., 2018). Hence, promoting adolescents’ prosocial behavior is of particular significance in that it lays a solid foundation not only for promoting their socialization but also for building a harmonious society (Carlo & Padilla-Walker, 2020; Kidron & Fleischman, 2006).

A large body of studies has explored the influencing factors of prosocial behavior, such as environmental factors and individual factors. In consideration of environmental factors, a substantial body of literature has demonstrated the important influence of family socioeconomic status (SES) on prosocial behavior. Andreoni et al. (2021) found that individuals with high SES have more prosocial behavior than those with low SES, which was contrary to findings of the negative relation between SES and prosocial behavior (Guinote et al., 2015). Parenting behaviors also play a crucial role in shaping adolescents’ psychological outcomes. Recent evidence highlights that not all forms of parental involvement are beneficial; for example, parental interference has been linked to increased depressive symptoms and burnout (Giacomo et al., 2026). Similarly, parenting styles and parental involvement predicted adolescent-reported academic burnout and engagement (Q. Zhu et al., 2023). However, individuals in mid-adolescence with consistent parental warmth were likely to benefit the most in terms of increased prosocial behavior (Buckley et al., 2024). Using a cross-lagged design, Luengo Kanacri et al. (2017) revealed that a positive school climate predicted prosocial behavior among adolescents over time; that is, adolescents perceiving more positive school climates tended to show more prosocial behavior in the following year. As an important environmental factor, social support is believed to be an important motivator of long-term prosociality (Trombini et al., 2025). Adolescents with higher levels of social support tend to exhibit more prosocial behavior and lower antisocial behavior (Liu et al., 2021). By providing emotional, informational and practical support, social support contributes to better psychological and physical health, ultimately guiding individuals’ behavioral responses.

Taking account of individual factors, researchers investigated the relationship between gender and prosocial behavior. In a meta-analysis study conducted by Xiao et al. (2019), small relative and absolute gender differences were found in adolescents’ prosocial behavior; females and males are generally more alike than different in their prosociality. Gratitude, as a positive social emotion, is also an individual factor that positively predicted prosocial behavior (Bartlett & DeSteno, 2006; L. K. Ma et al., 2017; N. Zhu et al., 2024). Gratitude is an important motivator for prompting the beneficiary to implement prosocial behaviors towards strangers even in uncertain situations, and could improve the quality of the relationship between the beneficiary and the helper. Individuals with higher levels of gratitude may behave more prosocially, be more caring about others, and also experience higher levels of happiness in their daily lives (Y. Ma et al., 2022; Oguni & Ishii, 2024). Experimental research also provides evidence that situational activation of self-awareness can increase willingness to engage in helping behavior (Scaffidi Abbate & Ruggieri, 2008). In addition, another important individual factor, personality, refers to the inner features of a person, which represents relatively stable attitudes and behavioral patterns towards environments. Applying the big-five factor personality model, researchers investigated the relation between five traits (conscientiousness, openness, agreeableness, extraversion, and neuroticism) and prosocial behavior, finding mixed or even contradictory results. For example, agreeableness was known to be the most closely associated with prosocial behavior (Habashi et al., 2016; Thielmann et al., 2020). Likewise, in a meta-analysis study, Kline et al. (2019) found that openness and agreeableness were significantly positively associated with prosocial behavior, while the other three big-five traits were not. However, Tariq and Naqvi (2020) indicated that conscientiousness, openness, agreeableness, and extraversion were positive predictors of prosocial behavior among adolescents, while neuroticism had been found to be negatively correlated with prosocial behavior.

Based on the theory of social behavior, as a positive social behavior, prosocial behavior is determined by multiple factors and is the result of the interaction of individual characteristics and situational factors (Staub, 2013). Although previous studies have explored the factors influencing prosocial behavior, most of which have mainly paid attention to either environmental or individual factors, to date, few have focused on the effect of their interactions on adolescents’ prosocial behavior. Thus, to address this knowledge gap, the present study examines the combination effect of these two factors from an integration perspective. To be specific, this study extended previous work and examined the relationship between conscientiousness and prosocial behavior among Chinese adolescents, and whether this relation depends on the indirect effect of empathy (an individual factor) and the moderating effect of social support (an environmental factor). Such understanding of the internal psychological mechanism between conscientiousness and prosocial behavior could provide enlightenment for developing effective interventions for cultivating prosocial behavior among middle adolescents.

### 1.1. Conscientiousness and Prosocial Behavior

According to the Big Five Taxonomy of traits, conscientiousness is one of the domains of personality traits (Goldberg, 1993), usually referring to individual differences in the propensity to regulate behaviors. For oneself, this means to have clear goals and plans, to have self-discipline, and to do things in an orderly and persevering manner, and this also means being responsible to others and society, manifested through adherence to social rules and norms, interpersonal responsibility and normative conformity (Bogg & Roberts, 2004; Lindahl, 2023; Roberts et al., 2014). Conscientiousness plays an important role in people’s work and life. Conscientiousness is not only a positive predictor of domain achievements such as academic performance for adolescents (Conrad & Patry, 2012), but also a reliable predictor of work performance for adults (Bakker et al., 2012; Byrne et al., 2005). It is plausible that individuals with higher conscientiousness may have better self-management capabilities related to goal achievement, time management, study and work performance, and be more willing to exert effort, pursue success and maintain self-discipline, which are the main determinants of gaining more domain achievements, including obtaining good school scores, receiving higher income, and holding leadership positions (Meyer et al., 2024; Sari, 2020; Saman & Wirawan, 2021; Verbree et al., 2023; Vianello et al., 2010).

A few empirical studies have demonstrated that conscientiousness or responsibility significantly predicts prosocial behavior (Afolabi, 2013; Alfirević et al., 2023; Pastor et al., 2024). For example, an experimental study revealed that compared with adolescents scoring lower for conscientiousness, adolescents reported to have higher conscientiousness showed a greater sense of obligation to help others and a greater likelihood of providing assistance in the help-eliciting prime condition (Swickert et al., 2014). De Groot and Steg (2009) showed that responsibility increased feelings of moral obligation, then induced prosocial behavioral intentions and actions. Xia and Yang (2020) proposed that highly conscientious individuals exhibit a heightened altruistic orientation and are more likely to engage in prosocial behaviors that benefit others. In contrast, when an individual has a lower level of conscientiousness, this predicting effect for prosocial motivation was weakened. A more recent study also found that adolescents with higher social responsibility were more capable of adjusting their behaviors based on moral obligations and values to meet social expectations, thereby engaging in more prosocial behaviors (Q. Li et al., 2024). These results support the Norm Activation Model of prosocial behavior theory, in which internalized social norms are the motivational factor for prosocial behavior, while individuals’ responsibility can activate social norms and generate a sense of moral obligation based on specific situations, hence potentially leading to prosocial behavior (Schwartz, 1973, 1977; Schwartz & David, 1976).

### 1.2. Indirect Effect of Empathy

Regarding empathy, although it has extensive history, it lacks a well-unified defined notion; different scholars have different definitions. In the current study, empathy means context-dependent emotional resonance that occurs when one shares the emotional state, situational understanding, and expectations of others, including both cognitive and affective components and depending on the interaction between trait capacities and state influences (Cuff et al., 2016; Eisenberg et al., 1999; Spinrad & Eisenberg, 2014). Empathy is considered to be an important determinant of prosocial behavior, which enables people to put themselves in others’ shoes and experience their emotions, ultimately leading to kind behaviors (Eisenberg & Fabes, 1990). It has been well established that there is a positive relation linking experiencing empathy and behaving prosocially (Eisenberg et al., 2010; W. Fu et al., 2022; Kamas & Preston, 2021; Telle & Pfister, 2016; Sze et al., 2012). Empathic emotion can promote the development of harmonious interpersonal relationships, stimulating prosocial motivation with the aim of improving others’ welfare (Carrizales et al., 2021; Yin & Wang, 2023). Experiencing positive empathy could elicit a motivation that might induce a positive emotional response, promoting prosocial behaviors which serve to maintain and prolong the experienced pleasant affective state (Telle & Pfister, 2016). Likewise, longitudinal studies have found that adolescents’ empathy predicted their prosocial behavior over time, and interventions or programs promoting empathy may contribute to increases in prosocial behavior among adolescents (Carrizales et al., 2025; Marshall et al., 2020; Yoo et al., 2013). In addition, experimental studies also demonstrated that this empathic concern produces altruistic motivation and evokes a selfless desire to improve the welfare of others, regardless of personal emotional rewards (Batson et al., 1991; Batson et al., 2015).

It is worth noting that conscientiousness is shown to be positively associated with empathy and is identified as an important predictor of empathic responding (Guilera et al., 2019; Melchers et al., 2016; Park & Lee, 2020). For instance, Song and Shi (2017) examined the relation between big-five personality traits and empathy among Chinese undergraduate medical students, and found that conscientiousness was modestly and positively associated with empathy, such as through perspective taking. Wan et al. (2019) also provided evidence that conscientiousness was significantly and positively associated with empathy capability in clinical nurses. This may be due to individuals with higher levels of conscientiousness usually having more responsibilities, which implies awareness of duties and obligations, so they tend to pay more attention to the unfavorable situations of others and have empathetic experiences (Abdullah et al., 2020; Chia & Tan, 2024; Suazo et al., 2020; Zahn-Waxler & Robinson, 1995). However, existing studies on the relationship between conscientiousness and empathy have primarily focused on healthcare professionals, with limited attention given to adolescents. In fact, considering the greater plasticity of personality traits during adolescence, more emphasis should be placed on adolescents’ conscientiousness, which is a key component in taking others’ perspectives, understanding their emotions, and ultimately guiding prosocial decisions and actions that benefit others or society.

### 1.3. Moderating Effect of Social Support

The social support theory posits that social support contributes to individual psychological development by offering emotional, informational, and practical assistance (Lakey & Cohen, 2000). Social support not only helps alleviate environmental stressors but also encourages positive behaviors and enhances proactive responses to environmental challenges (Cohen & McKay, 2020; Cohen & Wills, 1985; Y. Wang et al., 2024). Numerous empirical studies have further corroborated this perspective (Esparza-Reig et al., 2022; W. Wang & Wu, 2020; Yao & Li, 2023; You et al., 2022). Peng et al. (2023) indicated that social support was significantly and positively correlated with prosocial behaviors and directly influenced prosocial behaviors of vocational school students. Trombini et al. (2025) examined social support as a predictive factor for prosocial behavior through four studies, and the results showed that social support predicted prosocial activities several years later and fostered long-term prosocial behavior.

Previous studies have demonstrated that greater conscientiousness is associated with greater perceived social support (Barańczuk, 2019; Hill et al., 2014; Yu et al., 2021). When others are in need of help, a strong social support network can inspire a sense of responsibility, facilitate empathetic experience, stimulate the motivation to provide help, and thus lead to prosocial behavior (Calandri et al., 2019; Guariglia et al., 2023). However, when individuals have weaker social support systems, they usually tend to hold a negative and pessimistic attitude, experience more negative emotions, and display more problem behaviors (Javed et al., 2025; Younis et al., 2021). It is plausible that when individuals have close social relationships, they are more likely to be taken care of, which makes them more generous and willing to help others. In contrast, individuals with lower levels of social support are more prone to interpreting their environment negatively, perceiving the world as hostile and responding accordingly. Building on the social support theory and empirical evidence, it is important to understand the role of social support in the relation between conscientiousness and prosocial behavior among adolescents.

### 1.4. The Present Study

**Figure 1:**
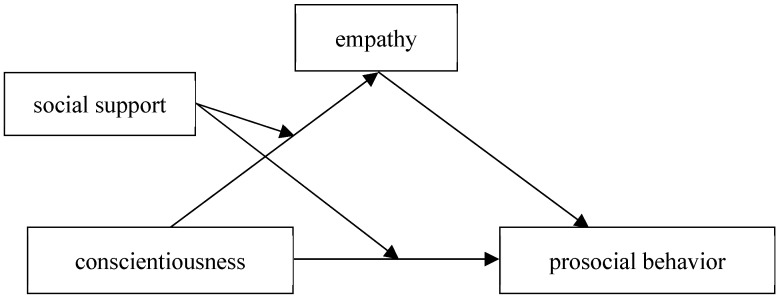
Hypothesized moderated model of mediation among conscientiousness, empathy, social support and prosocial behavior.

Based on the above theories and empirical studies, in the present study, we propose the following hypotheses and examine the psychological mechanism of the effects of conscientiousness, empathy, and social support on prosocial behavior among adolescents by constructing a moderated mediation model (see Figure 1).

**Hypothesis** **1.** *Conscientiousness is positively correlated with prosocial behavior among adolescents.*

**Hypothesis** **2.** *Empathy mediates the relationship between conscientiousness and prosocial behavior among adolescents.*

**Hypothesis** **3.** *Social support moderates both the direct relationship between conscientiousness and prosocial behavior and the indirect relationship between conscientiousness and prosocial behavior mediated by empathy. Specifically, at higher levels of social support, both the direct relation and indirect relation between conscientiousness and prosocial behavior are stronger than those observed at lower levels of social support.*

## 2. Methods

### 2.1. Participants

In the current study, a total of 1081 middle school students and high school students were selected from Zhejiang, Sichuan and Shanxi Provinces, China, including 531 boys (49.12%) and 550 girls (50.88%). Their age ranged from 13 to 18 years (*M* = 15.45, *SD* = 1.91). Due to practical constraints, a convenience sampling method was employed. Schools in these three provinces, representing different geographical regions and levels of economic development in China, were approached for this study. These schools had existing collaborations with our research team. Within each province, two schools were selected, and three to four classes were chosen from each school and all students in these classes were invited to participate.

After obtaining consent from the school administrators, parents, and the students, the participants were asked to complete the questionnaires through an online system in the class. A total of 1081 questionnaires were distributed and returned. After screening for invalid responses (e.g., incomplete questionnaires, patterned answering), 16 invalid questionnaires were excluded via listwise deletion. Finally, a total of 1065 valid questionnaires were obtained in this study (valid response rate = 98.5%).

### 2.2. Measures

#### 2.2.1. Conscientiousness

Conscientiousness was measured using the Chinese Big Five Inventory-2 (BFI-2) (Zhang et al., 2022). While the full BFI-2 consists of 60 items measuring five dimensions—extraversion, agreeableness, conscientiousness, neuroticism, and openness—the present study employed only the 12 items comprising the conscientiousness domain. The conscientiousness scale consists of three dimensions, each containing four items. Sample items were as follows: “Do things in an organized and systematic way” (organization), “Aim for efficiency and make sure to bring every undertaking to a successful conclusion” (productiveness), and “A reliable individual who consistently commands the trust of others” (responsibility). All items were scored on a 5-point Likert scale, ranging from 1 (strongly disagree) to 5 (strongly agree). Total scores were calculated by summing the 12 item scores. Higher total scores represent higher conscientiousness. The Cronbach’s alpha coefficient of the scale in this study was 0.86. The results of the CFA for this scale indicated acceptable fit indices: χ^2^/df = 2.06, *p* < 0.001, RMSEA = 0.06, SRMR = 0.04, CFI = 0.96, and TLI = 0.95.

#### 2.2.2. Empathy

The revised Chinese version of the Basic Empathy Scale (BES) was employed to measure empathy (Jolliffe & Farrington, 2006), which has been empirically validated in Chinese adolescents (C. Li et al., 2011). The scale consisted of 20 items across two dimensions: cognitive empathy (9 items) and affective empathy (11 items). An example of a cognitive empathy item is “When my friend performs excellently and achieves success, I can share his/her joy”, and an example of an affective empathy item is “I am highly susceptible to being influenced by the emotions of others”. The scale was scored using a 5-point Likert scale, ranging from 1 (strongly disagree) to 5 (strongly agree). Total scores were calculated by summing the 20 corresponding item scores. The higher the total scores, the higher empathy the participants had. A higher score for an individual indicates higher empathy. The Cronbach’s alpha coefficients for cognitive empathy and affective empathy were 0.87 and 0.89, respectively. A CFA in the present sample indicated acceptable fit indices: χ^2^/df = 1.77, *p* < 0.001, RMSEA = 0.05, SRMR = 0.05, CFI = 0.97, and TLI = 0.95.

#### 2.2.3. Social Support

The Perceived Social Support Scale (PSSS) was used to assess adolescents’ social support, and was revised in the Chinese sample based on Zimet’s Perceived Social Support Scale (Jiang, 1999; Zimet et al., 1990). The scale contains 12 items across three dimensions: family support (4 items; e.g., “My family can offer me tangible and targeted support”), classmate support (4 items; e.g., “I can count on my friends when confronted with troubles”), and others’ support (4 items; e.g., “When I encounter challenges, certain individuals, including my supervisors, relatives, and colleagues, will present themselves beside me”). Each item was rated on a 7-point Likert scale ranging from 1 (strongly disagree) to 7 (strongly agree). The total score was obtained by summing all 12 items. Higher total scores represented higher levels of social support. The Cronbach’s alpha coefficient for this scale in the present study was 0.92. All the fit indices of CFA for this scale were acceptable: χ^2^/df = 1.63, *p* < 0.001, RMSEA = 0.06, SRMR = 0.04, CFI = 0.98, and TLI = 0.96.

#### 2.2.4. Prosocial Behavior

Prosocial behavior was measured using the Adolescents Prosocial Tendencies Measure (Kou et al., 2007), which was revised in the Chinese adolescent sample based on the Prosocial Tendencies Measure (PTM) (Carlo & Randall, 2002), with a total of 26 items. The scale consists of six subscales: Public (4 items), Compliant (5 items), Altruism (4 items), Anonymous (5 items), Dire (3 items), and Emotional (5 items). Sample items were as follows: “In many public settings, I demonstrate a greater willingness to assist others” (Public), “I consistently respond promptly to requests for assistance without hesitation” (Compliant), “I donate money and goods without the intention of gaining any personal benefits” (Altruism), “I prefer to donate money anonymously” (Anonymous), “I tend to help people who are seriously injured or ill” (Dire), “I often help others when they are experiencing significant emotional distress” (Emotional). Adolescents evaluated their prosocial behavior on a 5-point Likert scale ranging from 1 (Does not describe me at all) to 5 (Describes me greatly). The total score was obtained by summing all 26 items. Higher total scores indicated more prosocial behavior. The Cronbach’s alpha coefficient for this scale in the present study was 0.90. The CFA for this scale indicated acceptable fit indices: χ^2^/df = 1.48, *p* < 0.001, RMSEA = 0.05, SRMR = 0.04, CFI = 0.96, and TLI = 0.96.

### 2.3. Procedure

This study was approved by the Ethics Committee of the authors’ university. Permission was also obtained from the participating schools. Prior to data collection, the researchers obtained informed consent from the participants in advance. Data were collected during regular school hours. On the day of the survey, students were informed that participation was completely voluntary and that they could withdraw at any time without any penalty. To ensure anonymity, no identifying information (e.g., names or student IDs) was collected. With the assistance of the schools’ computer teachers, participants completed an online questionnaire in school computer rooms under the supervision of trained research assistants. The computer teachers were present only to provide technical support and were not involved in administering the survey or interacting with students. Before the survey, the researchers explained the purpose of the study, emphasized that there were no right or wrong answers, and assured students of the confidentiality of their responses.

Participants completed online measures of conscientiousness, empathy, social support, and prosocial behavior. The average completion time was approximately 30 min. After finishing the questionnaire, each participant received a small gift as a token of appreciation. The gift was distributed after participation regardless of whether the student completed the survey.

### 2.4. Data Analysis

Pearson correlation analysis was employed to analyze the relationship between variables. Hayes’ PROCESS macro Model 4 was utilized to examine the mediating effect of empathy in the relationship between conscientiousness and prosocial behavior (Hayes, 2017). Conditional process analysis in the PROCESS macro Model 8 was used to test whether social support moderated this mediation process. The bootstrapping methodology (bootstrap sample = 5000) with 95% confidence intervals (95% CI) was applied for testing this moderated mediation effect (Igartua & Hayes, 2021). If the 95% confidence intervals did not include zero, the moderated mediation effect was significant. All variables were standardized and the demographic variables were controlled during statistical analysis. All analyses in the present study were conducted using SPSS 27.0.

## 3. Results

### 3.1. Correlation Analyses

Means, standard deviations, and bivariate correlations among key study variables are presented in Table 1. Prior to analysis, all continuous variables (conscientiousness, empathy, social support, prosocial behavior) were standardized as z-scores to facilitate interpretation of interaction effects in subsequent regression models.

As shown in Table 1, conscientiousness was negatively correlated with gender (*r* = −0.083, *p* < 0.01) and positively correlated with grade (*r* = 0.174, *p* < 0.01). Empathy was positively correlated with grade (*r* = 0.125, *p* < 0.01) and conscientiousness (*r* = 0.407, *p* < 0.01). Social support was negatively correlated with grade (*r* = −0.096, *p* < 0.01), and positively correlated with conscientiousness and empathy (*r* = 0.211, *p* < 0.01 and *r* = 0.196, *p* < 0.01, respectively). Prosocial behavior was positively correlated with conscientiousness (*r* = 0.348, *p* < 0.01), empathy (*r* = 0.271, *p* < 0.01) and social support (*r* = 0.614, *p* < 0.01).

### 3.2. Mediating Analysis

Based on prior research documenting gender differences in prosocial behavior (Eisenberg, 2006), grade-related developmental changes in empathy and social support (Malti et al., 2016; M. T. Wang & Eccles, 2012), and potential school environment factors on adolescents’ social behavior (Luengo Kanacri et al., 2017), we included gender, age, grade, and school as covariates in both the mediation and moderated mediation analyses. After controlling for these covariates, mediation analysis was subsequently utilized to explore the influence of empathy on the relationship between conscientiousness and prosocial behavior in middle school students (see Table 2). The results demonstrated a significant positive direct effect of conscientiousness on prosocial behavior (*β* = 0.35, *p* < 0.001), indicating that students with higher conscientiousness exhibited more prosocial behaviors. It also had a significant positive effect on empathy (*β* = 0.41, *p* < 0.001). When empathy as a mediator entered into the mediation model, conscientiousness also had a significant positive effect on prosocial behavior (*β* = 0.29, *p* < 0.001). The mediator empathy was found to significantly positively predict prosocial behavior (*β* = 0.15, *p* < 0.001), and the predictive indirect effect was statistically different from zero (*β* = 0.063, *p* < 0.001, 95% CI = [0.027–0.102]), indicating that empathy partially mediated the relationship between conscientiousness and prosocial behavior. The mediation effect accounted for 18% of the total effect. This result suggested that compared to students with lower conscientiousness, students who had higher conscientiousness were prone to experiencing higher empathy, which could lead to more prosocial behavior.

### 3.3. Moderated Mediation Analysis

**Figure 2:**
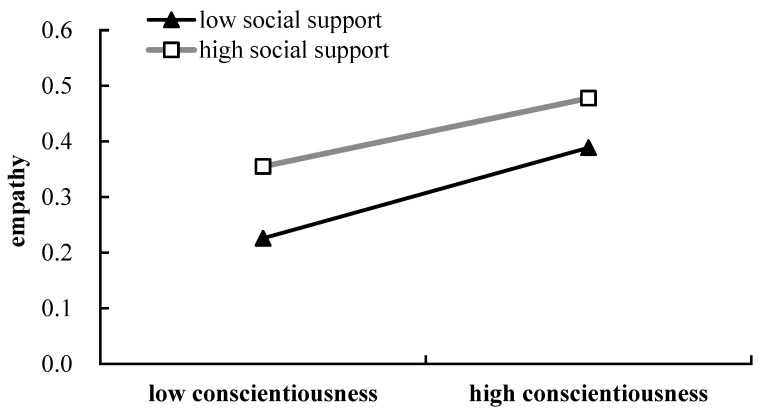
Social support moderates the relation between conscientiousness and prosocial behavior via empathy.

The moderated mediation analysis revealed that conscientiousness was a statistically significant predictor of both empathy (*β* = 0.362, *p* < 0.001) and prosocial behavior (*β* = 0.178, *p* < 0.001). Social support was also found to statistically positively predict empathy (*β* = 0.114, *p* < 0.001), and empathy also significantly and positively predicted prosocial behavior (*β* = 0.076, *p* < 0.01). There was a significant moderating effect of social support on the association between conscientiousness and empathy (*β* = 0.055, *p* < 0.05, 95% CI = [0.012–0.097]). This indicated that social support moderated the relationship between conscientiousness and prosocial behavior via empathy (see Table 3). A bootstrap analysis was then performed to examine this indirect effect of conscientiousness on prosocial behavior via empathy. The results on this moderated mediation effect showed that for both low levels of social support (95% CI = 0.226 to 0.389) and high levels of social support (95% CI = 0.355 to 0.478) (see Table 4), conscientiousness significantly predicted prosocial behavior through empathy. The result of the simple slope test demonstrated that the relation between conscientiousness and empathy was stronger with higher levels of social support (*β* = 0.416, *t* = 13.29, *p* < 0.001) than with lower levels of social support (*β* = 0.307, *t* = 7.41, *p* < 0.001) (see Figure 2). That is, for students with higher conscientiousness, those who have high levels of social support reported higher empathy than those with low levels of social support. The underlying mechanism may be that higher social support fosters emotional security, which in turn allows individuals with greater conscientiousness to redirect their attentional focus from internal to external demands, thereby facilitating the expression of greater empathy.

**Figure 3:**
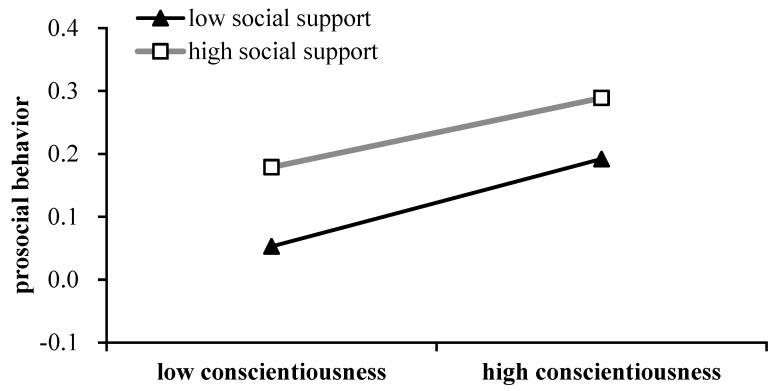
Social support moderates the relation between conscientiousness and prosocial behavior.

In addition, it should be noted that a significant interaction effect between the factors of conscientiousness and social support was observed (*β* = 0.056, *p* < 0.01, 95% CI = [0.020–0.091]), and this indicated that social support also moderated the direct relation between conscientiousness and prosocial behavior. A bootstrap analysis was then also performed to examine the conditional direct effects of conscientiousness on prosocial behavior. The results revealed that there were significant conditional direct effects not only for students with high social support (95% CI = 0.179 to 0.289) but also for students with low social support (95% CI = 0.053 to 0.192) (see Table 4). The simple slope analysis suggested that this conditional direct effect was stronger with high levels of social support (*β* = 0.233, *t* = 8.32, *p* < 0.001) and weaker with low levels of social support (*β* = 0.122, *t* = 3.47, *p* < 0.001) (see Figure 3). Our moderated mediation analyses for this conditional direct effect illustrated that for students with the same level of conscientiousness, those with high levels of social support reported more prosocial behavior than those with low levels of social support.

## 4. Discussion

The present study explored the relations between conscientiousness, empathy, social support and prosocial behavior among Chinese adolescents. The results demonstrated that all the relations between conscientiousness, empathy, social support and prosocial behavior were statistically significant. Conscientiousness was found to be positively correlated with prosocial behavior. Furthermore, empathy mediated the relationship between conscientiousness and prosocial behavior, while social support moderated not only the direct relationship between conscientiousness and prosocial behavior but also the indirect relationship between these variables mediated by empathy. Therefore, all the findings confirmed our initial hypotheses.

Our study confirmed the prediction of conscientiousness for prosocial behavior in adolescents: students with higher conscientiousness reported more prosocial behavior than students with lower conscientiousness, which was consistent with the findings of previous research (Patitsa et al., 2022; Shah & Rizvi, 2016; Trishala, 2021). Studies have shown that conscientiousness is a potent predictor of prosocial behavior which signifies obligations and responsibility, helps individuals regulate their values to comply with social norms, and promotes prosocial behaviors conforming to social requirements and expectations (Chia & Tan, 2024; Gungordu, 2023; Leng et al., 2020). It appears that students with higher conscientiousness tend to exhibit greater attention to others and society. They hope their actions can be beneficial to others and society, which fosters greater concern and enthusiasm for public affairs, thereby promoting increased prosocial behaviors such as engaging in community service or public welfare activities (Xie et al., 2016). On the other hand, students with lower conscientiousness usually focus exclusively on their own feelings and interests, neglecting to consider the circumstances of others, which diminishes the likelihood of prosocial behaviors.

Our study revealed that empathy mediated the relationship between conscientiousness and prosocial behavior among adolescents, which provided new evidence and enriched previous research. Previous studies have found a predictive effect of conscientiousness on empathy (Barrio et al., 2004; Doğan et al., 2025; Park & Lee, 2020; Wan et al., 2019). The predictive effect of empathy on prosocial behavior has also been strongly supported (Scaffidi Abbate et al., 2022; Telle & Pfister, 2016; W. Wang & Wu, 2020). Conscientiousness plays a crucial role in fostering an individual’s sense of responsibility to assist those in need, prompting greater attention to the feelings of others. It has been found that adolescents with high levels of empathy are often capable of emotionally placing themselves in others’ situations, genuinely experiencing the pain, anxiety, or predicament they face, which is associated with increased engagement in prosocial behaviors (Van der Graaff et al., 2018; Lee & Madera, 2021). According to the empathy–altruism hypothesis, through the perception of others’ emotional states and the subsequent generation of empathic concern, individuals are motivated to engage in selfless helping behaviors (Batson et al., 1991, 2015). Experimental studies have also demonstrated that when empathy levels are experimentally increased, individuals exhibit greater willingness to sacrifice personal interests to assist others and show enhanced tendencies to donate to strangers (Scaffidi Abbate & Ruggieri, 2008; Scaffidi Abbate et al., 2022; Sze et al., 2012).

As previously hypothesized, social support moderated the direct and indirect association between conscientiousness and prosocial behavior. Even though students reported lower levels of conscientiousness, those who received higher levels of social support still tended to report greater empathy and more prosocial behavior compared to their counterparts with lower social support. These results are in accordance with the social support theory. This theory posits that social support promotes individual social adaptation, facilitates better integration into society, fosters the development of healthy interpersonal relationships, and strengthens individuals’ sense of social responsibility and belonging, which lays a good foundation for the generation of prosocial behavior (Cohen & McKay, 2020; Cohen & Wills, 1985). Previous studies have found similar results (W. Fu et al., 2022; Trombini et al., 2025; Y. Wang et al., 2024).

According to trait activation theory (Tett & Burnett, 2003), social support may moderate the direct association between conscientiousness and prosocial behavior by facilitating the situational expression of conscientious traits in interpersonal contexts. Conscientiousness reflects a predisposition to be responsible, organized, and hardworking, but its expression in prosocial acts may depend on situational cues. A supportive social environment provides such cues—for instance, by creating social expectations of reciprocity, offering opportunities to help, or reinforcing prosocial norms. In this context, conscientious individuals are more likely to translate their sense of duty into actual prosocial behavior. Conversely, in low-support environments, even highly conscientious individuals may lack the contextual triggers or resources to act upon their prosocial intentions.

The moderating role of social support in the empathy-mediated indirect pathway between conscientiousness and prosocial behavior can be understood through the lens of Ecological Systems Theory (Bronfenbrenner, 1979), which emphasizes how relational contexts shape the activation and expression of individual traits and socioemotional processes. Firstly, receiving social support, particularly emotional support from friends, parents, and teachers, can help adolescents experience feelings of warmth and comfort, develop a strong sense of security, and perceive that they are being cared for (J. Li et al., 2021). This, in turn, motivates them to offer assistance to others in need, which may be conducive to the improvement of conscientiousness. Moreover, greater social support facilitates the development of higher empathy in adolescents, including enhanced perception of others’ emotions and an increased capacity to generate beneficial reactions to others’ situations (Chen & Xu, 2021). Additionally, social support can also provide adolescents with practical guidance, such as by encouraging their participation in volunteer services, community activities, and other forms of social engagement. The more external support they receive, the more positive emotional experiences they are likely to have, and the greater possibility to engage in external activities, thereby promoting the development of prosocial behavior more broadly (Esparza-Reig et al., 2022; Javed et al., 2025). Meanwhile, students lacking social support may experience perceived social exclusion and exhibit reduced empathic concern for others. When adolescents feel uncertain about their access to social support and resources, they are less likely to engage in prosocial behaviors or demonstrate a willingness to give back to others and society.

## 5. Limitations and Implications

The present study explored the psychological mechanisms that influence prosocial behavior in Chinese adolescents. There are several limitations that should be considered. First, our study was based on a cross-sectional survey design; due to the lack of temporal precedence, we could not make a causal inference from the mediation analysis on the relationship between conscientiousness and prosocial behavior. A longitudinal design should be employed to verify the causal relationships between these variables in future studies. Second, the data we collected in our study only used the self-reporting method, which could introduce measurement errors and potentially bias the structural relationships between constructs. Future research should consider collecting data using other evaluation methods (peer, parent and teacher reports) or interviewing methods to enhance the robustness of the findings. Third, the variables examined in our study correlated with prosocial behavior were limited. Future research should include other positive psychological characteristics (e.g., gratitude, agreeableness, etc.) to obtain a more comprehensive understanding of the development of adolescents’ prosocial behavior. Fourth, due to the limitations of our human resources, a limited number of students participated in our study, which may limit the generalizability of our findings. To strengthen the external validity of the proposed model, future studies should recruit participants from more regions in China and analyze the influence of this demographic information.

Despite the limitations of the present study, it retains significant research value and important educational implications. This study examined the relationships among conscientiousness, empathy, social support, and prosocial behavior through constructing a moderated mediation model. This model extends existing research by identifying the mediating role of empathy and the moderating effect of social support in the relation between conscientiousness and prosocial behavior. The findings have educational implications for the cultivation of prosocial behavior among adolescents. Firstly, based on the contribution of conscientiousness to prosocial behavior, parents and teachers should encourage adolescents to establish a sense of conscientiousness and guide them to care for others as well as respect and understand others. Secondly, the findings of the present study emphasize the mediation effect of empathy in the relation between conscientiousness and prosocial behavior, which should inspire parents and teachers to pay more attention to the cultivation of empathy in adolescents, fostering the ability to understand others’ perspectives and promoting more effective interpersonal interactions. Thirdly, the results indicated that social support moderates the relation between conscientiousness and prosocial behavior through empathy. This implies that a better social support network is beneficial to the development of prosocial behavior for adolescents. Parents and teachers should guide adolescents in participating in diverse forms of social engagement. When adolescents exhibit prosocial behaviors, educators should offer encouragement to reinforce the value of these prosocial behaviors.

## 6. Conclusions

The present study identifies a significant positive association between conscientiousness and prosocial behavior among adolescents. It further demonstrates that empathy mediates the relationship between conscientiousness and prosocial behavior, and that social support moderates both the direct path from conscientiousness to prosocial behavior and the indirect path mediated through empathy. This expands the literature on how individual traits shape prosocial behavior, providing empirically grounded insights for fostering prosocial development in adolescents. Future research should prioritize longitudinal designs or experimental manipulations to generate more robust evidence regarding causal associations between conscientiousness and prosocial behavior.

## Figures and Tables

**Table 1:** Bivariate correlations across main variables (*N* = 1065).

Variables	M ± SD	1	2	3	4	5	6
1. Gender	1.51 ± 0.50	-					
2. Grade	3.54 ± 1.55	−0.026	-				
3. Conscientiousness	38.04 ± 5.12	−0.083 **	0.174 **	-			
4. Empathy	62.44 ± 8.91	0.016	0.125 **	0.407 **	-		
5. Social support	61.24 ± 13.39	0.047	−0.096 **	0.211 **	0.196 **	-	
6. Prosocial behavior	94.04 ± 17.08	−0.050	0.016	0.348 **	0.271 **	0.614 **	-

Note: ** *p* < 0.01. Gender was coded as 1 = male, 2 = female.

**Table 2:** Results of mediation analysis.

Predictors	Model 1 (Prosocial Behavior)		Model 2 (Empathy)		Model 3 (Prosocial Behavior)	
	*β*(SE)	*t*	*β*(SE)	*t*	*β*(SE)	*t*
Conscientiousness	0.35(0.03)	12.11 ***	0.41(0.03)	14.55 ***	0.29(0.03)	9.16 ***
Empathy					0.15(0.03)	4.97 ***
*R* ^2^	0.12		0.17		0.14	
*F*	146.64 ***		211.65 ***		87.31 ***	
Indirect effect			B	Boot SE	LLCI	ULCI
Empathy			0.063	0.020	0.027	0.102

Note. Each variable is standardized prior to analysis. Abbreviations: SE, standard error; LLCI, lower limit confidence interval; ULCI, upper limit confidence interval. *** *p* < 0.001.

**Table 3:** Results of moderated mediation analysis.

Predictors	Model 1 (Empathy)			Model 2 (Prosocial Behavior)		
	*β*(SE)	*t*	95% CI	*β*(SE)	*t*	95% CI
Conscientiousness	0.362(0.029)	12.213 ***	0.304, 0.419	0.178(0.026)	6.778 ***	0.127, 0.230
Social support	0.114(0.028)	3.998 ***	0.058, 0.169	0.555(0.024)	23.353 ***	0.508, 0.602
Conscientiousness × Social support	0.055(0.022)	2.514 *	0.012, 0.097	0.056(0.018)	3.066 **	0.020, 0.091
Empathy				0.076(0.026)	2.958 **	0.025, 0.126
*R* ^2^	0.184			0.437		
*F*	79.523 ***			205.664 ***		

Note: Bootstrap sample size = 5000. Abbreviations: 95% CI, bootstrapped confidence intervals; SE, standard error. * *p* < 0.05. ** *p* < 0.01. *** *p* < 0.001.

**Table 4:** Bootstrap analysis of conditional indirect effects of conscientiousness on prosocial behavior via social support.

Condition	Social Support	Effect	BootSE	BootLLCI	BootULCI
Conscientiousness → Empathy → Prosocial behavior	M − 1SD	0.307	0.042	0.226	0.389
	M	0.362	0.030	0.304	0.420
	M + 1SD	0.416	0.031	0.355	0.478
Conscientiousness → Prosocial behavior	M − 1SD	0.122	0.035	0.053	0.192
	M	0.178	0.026	0.127	0.230
	M + 1SD	0.233	0.028	0.179	0.289

Abbreviations: SE, standard error; LLCI, lower limit confidence interval; ULCI, upper limit confidence interval.

## Data Availability

The datasets are not publicly available due to privacy protection concerns, but can be made available by the corresponding author on reasonable request.
